# Green Exercise: How Are Characteristics of Urban Green Spaces Associated with Adolescents’ Physical Activity and Health?

**DOI:** 10.3390/ijerph16214281

**Published:** 2019-11-04

**Authors:** Abdullah Akpınar

**Affiliations:** Department of Landscape Architecture, Faculty of Agriculture, Aydın Adnan Menderes University, Aydın 09100, Turkey; abdullah.akpinar@wsu.edu; Tel.: +90-256-772-7023 (ext. 6491)

**Keywords:** green exercise, urban green space, features, physical activity, health, BMI

## Abstract

This study investigates associations between characteristics of urban green spaces (UGSs) and adolescents’ self-reported green exercise (GE), general health, and body mass index (BMI). Data were collected through face-to-face personal interviews with 384 adolescents ages 13–19 between 1 March and 31 May, 2018 in UGSs in Aydın, Turkey. Multivariate regression analyses were conducted to examine associations controlling for confounding factors. Stratified analyses were also conducted to determine differences between boys and girls. Positive associations with the duration of GE included many trees, lawns, soccer fields and basketball courts, play equipment, and self-reported general health. Whereas increased BMI was associated with increased benches/ seating, lawns and exercise trails were positively associated with self-reported general health. Negative relationships with increased BMI included many trees, open areas, and outdoor fitness equipment. Increased distance from UGSs was negatively associated with the frequency of GE. In stratified analyses, positive associations between boys’ duration of GE and self-reported general health were found. Whereas soccer fields and basketball courts were associated with girls’ frequency of GE, exercise trails and play equipment were correlated with girls’ self-reported general health. Negative associations with the boys’ frequency of GE included increased BMI and screen time. Whereas increased distance from UGSs was negatively associated with girls’ frequency of GE, many trees, lawns, exercise trails, play equipment, open areas, flowerbeds, and outdoor fitness equipment were negatively correlated with girls’ increased BMI. Findings suggest that adolescents’ GE and health could be promoted with many trees, lawns, flowerbeds, open areas, play and outdoor fitness equipment, exercise trails, and soccer fields and basketball courts. Findings of this study should be tested with longitudinal or intervention studies in future research.

## 1. Introduction

Turkey’s rapid urbanization since 1980 has dramatically reduced outdoor play areas for children which, in turn, increased the risk for adiposity and physical inactivity among children and adolescents. While the obesity rate in 1995 in Turkey was only 2.6% and 1.55% for boys and girls respectively [1], it is today 8.4% [2]. The other worrisome issue for children and adolescents is physical inactivity which could lead to serious health problems such as psychological disorders, coronary heart disease, breast and colon cancers, chronic diseases, type 2 diabetes, adiposity, and shortens life expectancy [3,4]. According to the report of Turkish Ministry of Health, physical inactivity of children aged 6–11 and adolescents aged 12–18 in urban areas are 58.2% and 57%, respectively [1]. Physical inactivity of children and adolescents is a serious issue for adults since physical inactivity lifestyle of children and adolescents are associated with poor adult health outcomes [5,6].

Health benefits of physical activity (PA) for children and adolescents, on the other hand, have been well documented. PA during childhood and adolescence is associated with improving bone and skeletal health [7,8,9], cardiovascular health [10,11], motor skill development [12,13], cognitive functioning [14,15], and sleep quality [16]. PA in that period is also associated with reducing adiposity [17,18,19] and symptoms of depression and anxiety [20,21,22]. PA has also positive impacts on academic performance [23] and self-esteem [24]. In addition, developing active and healthy lifestyles during adulthood is supported by PA during childhood and adolescence periods [25,26].

Considering the prevalence and impacts of physical inactivity, it is crucial to increase adolescents’ PA. To do this, investigating the factors that affect adolescents’ PA is necessary. In this context, socio-ecological models are frequently used by public health researchers to understand the determinants of PA [27,28,29]. In this model, urban green spaces (UGSs) are considered one of the potential environmental factors that influence adolescents’ PA level [30,31,32]. For instance, a recent study conducted in the Netherlands revealed that in order for adolescents to visit UGSs most frequently, the presence of green spaces in the neighborhood was important [33]. Studies also showed that UGSs closeness to adolescents’ home was positively associated with increased PA levels [31,34]. Hence, access to UGSs was the most frequently reported factor to promote adolescents’ PA levels in related studies [30,35].

A growing body of studies suggest that nature provides opportunities for free exploration, hence, exposure to nature could positively affect adolescents’ health [36,37,38]. As previous studies suggested, PA in green spaces/natural environments, or green exercise (GE), provides significant physiological and psychological health benefits [39,40,41]. Studies, for instance, showed that GE was positively associated with blood pressure, heart rate [42], general health [31], mental health, and cognitive development of children and adolescents [38,43]. Studies also found that GE promotes self-discipline, attention restoration, memory, supportive social groups, competence, moderates stress, and improves behaviors while reducing risky behaviors [43,44]. GE also reduces adiposity of children and adolescents [45,46].

Although UGSs are important environmental factors for adolescents’ PA, some characteristics and features of UGSs could be more attractive or motivating than others for adolescents’ GE [47]. Previous studies showed that large open spaces [48], many trees [49], greenness [32,50], presence of nature [51], walking paths, picnic areas, shelters, BBQs and restrooms, skate parks, and pool and water features [49,52], play facilities, sport fields, and playgrounds/outdoor fitness equipment [48,52,53], well maintained parks [53,54], and attractive scenery [48] were significantly associated with adolescents’ visitation and GE. In addition, it is also found that body mass index (BMI) and environmental factors such as neighborhood environments, convenient facilities, etc., mediate adolescents’ GE, therefore, further investigation is recommended [47].

While benefits of GE on adolescents’ health are known, there is a gap in existing knowledge about how different characteristics of UGSs influence adolescents’ GE and shape their health. In a literature review, for instance, Gardsjord, Tveit, and Hordh [30] showed that a limited number of studies explored parks’ features and adolescents’ PA, concluding adolescence is an understudied age group. Likewise, current studies emphasized the dearth of studies about adolescents’ GE and suggested further inquiries for the better understanding of adolescents’ GE [48,49,55,56]. In addition, a lack of studies about adolescents’ GE and health from developing countries [30,34] restricts the generalization of the findings, which is an obstacle to creating a global framework for the design and use of UGSs [37,57]. Furthermore, inconsistent findings about gender differences and beneficial effects of GE on adiposity require further investigations [31,32,58].

The primary aim of this research was to investigate the associations between characteristics of UGSs and adolescents’ self-reported PA and health. The secondary aim of this study was to examine the gender differences among these relationships. Based on the research aims of the study, the following questions were explored:

1. What are the relationships between characteristics of UGSs and adolescents’ self-reported frequency and duration of GE?
2. What are the relationships between characteristics of UGSs and adolescents’ self-reported general health and BMI?
3. How do these relationships differ between boys and girls?

## 2. Materials and Methods

### 2.1. Study Sites and Participants

**Figure 1:**
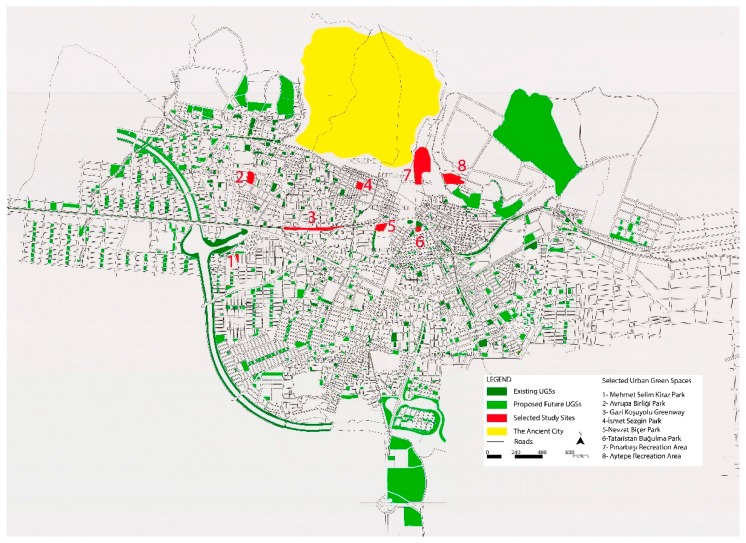
Selected study sites within the city limit [59].

The data used in this study were collected in eight different UGSs (i.e., a greenway, an urban park, neighborhood parks, and recreational areas) in Aydın, Turkey (Figure 1). These UGSs were chosen for the study due to their characteristic features, usage rates, locations, and neighborhoods’ socio-economic-status (SES). Adolescents’ self-reported participation in outdoor organized or unorganized play, exercise, or sport activities were defined as PA in the study. To carry out this research, first the ethics approval from the Ethics Committee of Aydın Adnan Menderes University was acquired (Protocol Number: 2018/1530). Then, the study sites were determined. After that, face-to-face personal interviews were conducted with 384 adolescents ages 13–19 between 1 March and 31 May, 2018. Permission from parents of those who agreed to participate in the study and were under 18 years of age were sought. Adolescents provided their demographic and SES such as gender, age, height, weight, education level, and monthly family income.

### 2.2. Questionnaire-Evaluation of UGSs

**Figure 2:**
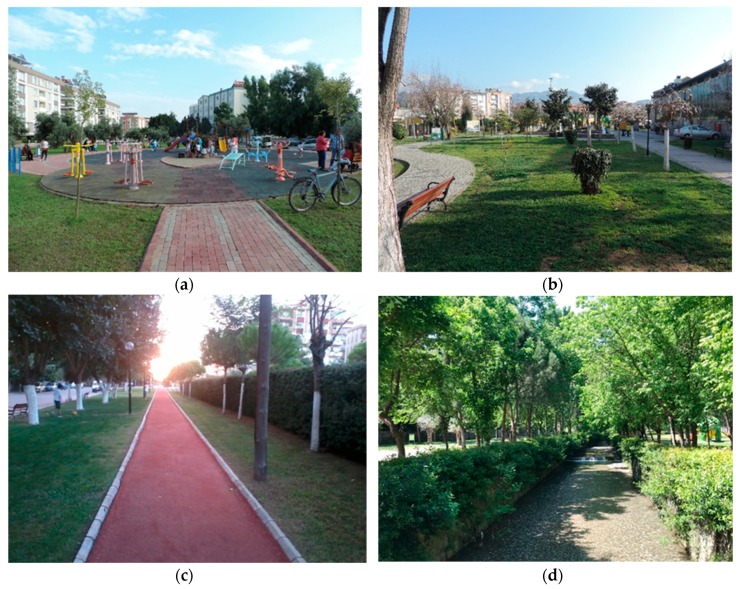
Some of the study sites where the data were collected (**a**) Mehmet Selim Kiraz Park, (**b**) Nevzat Biçer Park, (**c**) Gazi Koşuyolu Greenway, (**d**) Pınarbaşı Recreational Area.

UGSs were used to reach adolescents (Figure 2). Surveys were conducted during both weekdays and weekends visits which lasted 2–3 h in the morning from 7 to 9 a.m., in the midday from 12 to 2 p.m., and in the evening from 5 to 8 p.m. The target group was the general population of adolescents. First surveyors observed adolescents who were physically active or passive or passerby on UGSs, then explained to them what the study was about, and invited them to participate in the survey. Parents of those adolescents who agreed to participate in the survey and under 18 years of age were contacted to obtain the parents’ consent. The questionnaire asked adolescents for the demographic and SES, the walking distance from their home to the closest UGS, their frequency and duration of GE on a five-point Likert scale, and their screen time (i.e., on TV, computers, laptops, game consoles, etc.) per day. The respondents were also asked to evaluate their own general health considering the last two weeks using five-point Likert scales.

The characteristic features of UGSs (i.e., play equipment, outdoor fitness equipment, soccer fields and basketball courts, exercise trails, open areas, many trees, varied plants and animal life, lawn, flowerbeds, lights, picnic areas, and many benches/seating) were evaluated and rated by two professional landscape architects using five-point Likert scales from one to five (each score means different level of presence). For this evaluation, a qualitative approach was adopted where the characteristic features of UGSs were rated based on the experience and knowledge of two professional landscape architects [60,61].

### 2.3. BMI Measure

Based on the self-reported information provided by the adolescents about weight and height, their BMI was calculated. Adolescents’ BMI was calculated as weight in kilograms divided by height in meters squared (kg/m^2^). Then, adolescents’ percentile was calculated according to their age. In this calculation, percentile range of Centers for Disease Control and Prevention was used [62]. Percentile was broken into (a) less than the fifth percentile as underweight (coded 1), (b) fifth percentile to less than the 85th percentile (coded 2), (c) 85th to less than the 95th percentile as overweight (coded 3), and (d) equal to or greater than the 95th percentile as obese (coded 4).

### 2.4. Analytic Strategy

First, normality of the variables was checked by using the Kolmogorov–Smirnov test. The distributions of responses to frequency and duration of GE, general health, and BMI were normal. Variance inflation was also checked to detect multicollinearity issues. No multicollinearity issues between independent variables were found. Then, several multivariate linear regression analyses were performed to investigate the associations between characteristics of UGSs and (i) adolescents’ frequency and duration of GE and (ii) adolescents’ general health and BMI while controlling for confounding factors. In addition, stratification analyses were conducted to explore the differences between boys and girls. The results are presented as unstandardized coefficients (*b* and SE) with 95% confidence intervals (CI). A *p*-value of 0.05 was used to indicate statistical significance.

## 3. Results

### 3.1. Demographics and GE Status

The sample consisted of 384 participants aged between 13 and 19 years (M_age_ = 15.57 years, SD = 1.48). Fifteen years old participants represented a quarter of the participant group (25.3%). Boys represented just over half (55.2%) of the sample. BMI ranged from 12.11 to 33.30 (M_BMI_ = 21.58, SD = 3.07). Most participants (77.9%) were high school students. Monthly household income of adolescents varied from less than 999 Turkish Lira to more than 6000 or more Turkish Lira (Mdn_income_ = 2000–2999 Turkish Lira, which is just over the families’ monthly household income in Turkey [63]). Among all individuals, only 12.2% adolescents rated their general health as bad or very bad, which shows the data consists of self-reportedly healthy sample of adolescents. As seen in Table 1, overall just 9.6% of adolescents do daily GE, whereas 22.7% perform GE several times per week. For the duration of GE, overall 29.2% of adolescents spend more than 1 h participating in GE. Regarding gender, girls’ frequency of daily GE (11.0%) was higher than boys (8.5%), while more boys than girls spend more than 2 h participating in GE (11.3% and 4.1%, respectively). In terms of screen time, 26.1% spend more than 3 h screen time, while 31.7% spend less than 1 h for screen time. Comparing the screen time between gender, more boys (34%) spend greater than 3 h than girls (16.3%).

### 3.2. Associations between Characteristics of UGSs and Adolescents’ Frequency and Duration of GE 

A series of multivariate linear regression analyses were performed to explore the relationships between characteristics of UGSs and adolescents’ frequency and duration of GE while controlling for confounding factors (i.e., gender, age, education, income, BMI, and distance to UGSs from home). As seen in Table 2, the findings show that distance to UGSs from home (95% CI = −0.189 – −0.045) was negatively associated with adolescents’ frequency of GE, whereas many trees (95% CI = −0.071–2.021), lawns (95% CI = −0.049–2.241), play equipment (95% CI = −0.212–3.430), and soccer fields and basketball courts (95% CI = −0.016–0.605) were positively associated with adolescents’ frequency of GE. In terms of covariates, a positive significant association between age (95% CI = −0.016–0.605) and frequency of GE was found, whereas education level (95% CI = −0.016–0.605) was negatively associated with frequency of GE. In terms of duration of GE, no significant association was found between characteristics of UGSs and adolescents’ duration of GE. Regarding the covariates, the findings show that while boys (95% CI = −0.032–0.465) and income (95% CI = 0.033–0.201) were positively and significantly correlated with duration of GE, increased BMI (95% CI = 0.033–0.201) was negatively and significantly associated with duration of GE.

### 3.3. Associations between Characteristics of UGSs and Adolescents’ Self-Reported General Health and BMI 

A series of multivariate linear regression analyses were conducted to investigate the associations between characteristics of UGSs and adolescents’ self-reported general health and BMI while controlling for confounding factors. As shown in Table 3, the findings revealed lawns (95% CI = −0.149–1.797), exercise trails (95% CI = −0.060–0.578), and duration of GE (95% CI = 0.061–0.261) were positively and significantly associated with adolescents’ self-reported general health. In terms of covariates, a positive significant association between education level (95% CI = −0.096–0.797) and self-reported general health was found. Regarding BMI, the findings revealed negative significant relationships between many trees (95% CI = −4.765–0.349), lawns (95% CI = −4.904–0.700), exercise trails (95% CI = −1.314–0.177) and outdoor fitness equipment (95% CI = −8.319–0.578) and self-reported increased BMI. A positive significant relationship was found between many benches/seating (95% CI = −0.341–2.878) and self-reported increased BMI. Regarding the covariates, there were positive significant associations between boys (95% CI = 0.396–1.648) and income (95% CI = −0.069–1.949) and self-reported BMI.

### 3.4. Stratified Analyses of Gender Groups 

Stratification analyses were conducted to further examine differences among boys and girls. As seen in Table 4, no characteristics of UGSs were significantly correlated with boys’ frequency and duration of GE. Regarding the covariates, boys’ frequency of GE was positively correlated with age (95% CI = −0.016–0.281) and negatively associated with education level (95% CI = −1.106–0.018), increased BMI (95% CI = −0.107–0.021), and screen time (95% CI = −0.194–0.014). For girls, distance to UGSs from home (95% CI = −0.268–−0.049) was negatively associated with girls’ frequency of GE, while soccer fields and basketball courts (95% CI = 0.016–1.016) were positively correlated with girls’ frequency of GE. In regard to covariates, while education level (95% CI = −1.267–0.000) was negatively associated with girls’ frequency of GE, income (95% CI = −0.025–0.275) was positively correlated with girls’ duration of GE.

In terms of general health, stratified analyses revealed that no characteristics of UGSs were significantly associated with boys’ self-reported general health and BMI (Table 5). While boys’ duration of GE (95% CI = 0.033–0.314) and education level (95% CI = 0.025–1.006) were positively associated with self-reported general health, age (95% CI = −0.250–0.008) and screen time (95% CI = −0.429–0.024) were negatively associated with self-reported general health and BMI, respectively. For girls, the findings revealed that duration of GE (95% CI = −0.020–0.283), lights (95% CI = −0.007–3.487), exercise trails (95% CI = −0.069–0.838), and play equipment (95% CI = −0.249–4.547) were positively associated with girls’ self-reported general health. In contrast, the findings showed that many trees (95% CI = −5.251–−0.091), lawns (95% CI = −5.580–−0.742), exercise trails (95% CI = −2.562–−0.071), play equipment (95% CI = −5.472–−0.923), open areas (95% CI = −3.759–−0.299), flowerbeds (95% CI = −2.912–−0.366), and outdoor fitness equipment (95% CI = −2.204–−0.771) were negatively correlated with girls’ increased BMI.

## 4. Discussion

**Figure 3:**
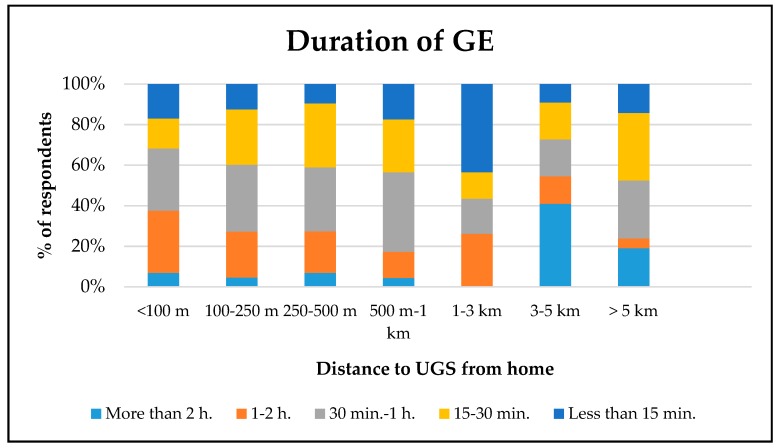
Distance to UGS versus duration of GE, in percent of the respondents. GE: Green exercise, UGS: urban green space.

As previous studies reveal, physical factors of UGSs may influence the use and engagement with nearby green spaces [64]. Supporting the previous studies, this study reveals that some characteristic features of UGSs influence adolescents’ GE and health. In general, the findings show that increased distance from UGSs was negatively associated with adolescents’ frequency of GE. This finding is in line with previous studies [31,32,34] and proximity seems to play a greater role in promoting GE among adolescents. However, proximity alone may not be enough to promote adolescents’ GE. As seen in Figure 3, while distance to UGSs from home increases, the duration of GE does not consistently decrease, which indicates some other factors may have influenced adolescents’ GE. Supporting the notion, the findings show that many trees, lawns, and soccer fields and basketball courts were positively associated with duration of GE. These findings are consistent with previous studies as it seems these characteristics encourage adolescents to spend longer time for GE [30,49,52,53]. For instance, Van Hecke, et al. [53] found that a park with a soccer field and a basketball court was preferred by adolescents compared to a park without a soccer field and a basketball court. This is expected since soccer fields and basketball courts provide PA opportunity for adolescents [49,52]. Trees and lawns were also found to be important characteristics of UGSs to promote adolescents’ GE. Previous studies showed that well-maintained lawns make people feel safer [65,66], while trees make people relax and create zones of shelter [30]. The findings of the study suggest that if these characteristic features are present in UGSs, it is more likely that adolescents will be more physically active. Therefore, policymakers and landscape architects should ensure that UGSs have many trees, well-maintained lawns, and soccer fields and basketball courts to promote adolescents’ GE in UGSs.

In terms of health, the findings show that duration of GE, lawns, and exercise trails were positively associated with adolescents’ self-reported general health. While increased benches/seating were positively associated with increased BMI, many trees, open areas, outdoor fitness equipment were associated with lower healthier range of BMI. The finding about the positive relationship between adolescents’ GE and self-reported general health is consistent with the previous studies [31,43,67], which suggests that GE could provide health benefits to adolescents. In contrast, no studies investigating the relationships between characteristics of UGSs and adolescents’ self-reported general health and BMI were found in the literature review to compare the results. Nonetheless, possible explanations have been discussed. Studies showed that UGSs’ greenness was found to be associated with adolescents’ GE [32,50]. Trees and grasses are the main components of greenness in UGSs. It is probable that these characteristics attract and encourage adolescents for participating in GE, which promotes a better general health and lower BMI. Similarly, exposure to UGSs has been associated with lower BMI [68] and a higher level of PA [30,69]. As previous studies show, open areas, walking paths/exercise trails, and outdoor fitness equipment are positively associated with GE among children and adolescents [48,52,53]. These characteristics feature in UGSs most likely provide opportunities for adolescents to exercise, which improves adolescent’ general health and reduces adiposity. The findings also revealed that increased benches/seating were positively associated with increased BMI. Similarly, a recent study about park characteristics and adolescents’ park-based PA found that adolescents would rather use a park without benches/seating when they engage in GE [53]. A possible explanation for this finding is that adolescents may feel encouraged to sit when they see benches/seating instead of engaging in GE [53], which may have resulted higher BMI among adolescents. Considering the results of the study, the findings suggest that providing UGSs having many trees, well-maintained lawns, open areas, exercise trails, and outdoor fitness equipment could be an effective strategy to improve adolescents’ general health and reduce adiposity.

The findings also showed that while education level was significantly associated with frequency of GE, no significant association was found for age. The possible explanation for this outcome could be the dropout of Turkish students. Studies show that while the compulsory education in Turkey is 12 years, dropout rate increases at the age between 15 to 19 among adolescents [70]. Therefore, this may have affected the findings of the study. The time pressure is another possible explanation for the negative significant association for the education level. In Turkey, adolescents are under pressure for academic success and only 35% can enter the university after taking the University Entrance Exam [71]. Students have to take private courses after school hours and during the weekends, which may negatively affect their PA level. 

More detailed analyses were conducted to determine the differences between boys and girls. The findings revealed that while no characteristics of UGSs were significantly associated with boys’ frequency and duration of GE, their increased BMI and screen time were negatively associated with frequency of GE. Some of the findings are in line with previous studies, while some are not consistent with previous studies. As previous studies show, boys tend to be more physically active than girls [31,72,73]. At the same time, boys spend more time on the screen than girls [64]. It is also seen in Table 1 that boys have higher screen time (i.e., more than 3 h) than girls. It seems that boys higher PA reduces their adiposity while screen time negatively influence boys GE. For girls, increased distance to UGS was negatively associated with girls’ duration of GE, which could be related to safety concerns. In previous studies, concern for safety was one of the important barriers for girls to visit UGSs and engage in GE [74]. It is probable that parents feel safe when their daughters engage in GE in UGSs close to home and let them play longer [31,74]. On the other hand, the findings show an interesting result that the association between soccer fields and basketball courts was evident for girls but not for boys. This finding suggests that presence of soccer fields and basketball courts was also important for girls to engage in GE [75]. Considering no characteristics of UGSs were associated with both boys and girls GE (i.e., except soccer fields and basketball courts), these findings are unexpected considering previous studies [48,49,50,53]. A possible explanation for these unexpected results could be that UGSs may not have addressed the needs of boys and girls in terms of quality and design. As previous studies show, adolescents may not visit UGSs if they consider UGSs designed for younger children, not challenging enough, or playground equipment uninteresting [55,76]. These possibilities need to be investigated in future studies.

Regarding health findings, the results reveal that boys’ duration of GE was positively associated with their self-reported general health. For girls, exercise trails and play equipment were positively correlated with girls’ self-reported general health, while many trees, lawns, flowerbeds, open areas, exercise trails, play equipment, and outdoor fitness equipment were negatively associated with girls’ increased BMI. These findings support the previous studies that spending longer time in UGSs could provide health benefits [31,38,43]. It is also likely that open areas with many trees, lawns, and flowerbeds increase the attractiveness of UGSs and trails, play and exercise equipment provide opportunity to engage in GE for girls [49,75]. These possibilities and why no characteristics of UGSs were associated with boys’ self-reported general health and BMI warrant further research. Therefore, further investigation on this issue is recommended.

To the author’s knowledge, this study is one of the first examinations of the relationships between characteristics of UGSs and adolescents’ self-reported general health and BMI. The other strength of this paper is that instead of interviewing with parents, face-to-face personal interviews were conducted with adolescents. On the other hand, some limitations should be acknowledged. The primary limitation of this paper is that it was cross-sectional study rather than longitudinal or intervention studies. However, while causal relationships cannot be drawn, this cross-sectional study of the relationship of UGS and adolescents’ health adds to current knowledge, which provides data for future longitudinal or intervention studies. While the data of the study was reported by adolescents themselves, it still depends on their recall. Hence, the data was prone to recall bias. Social desirability could have affected the response of the adolescents as well. They might have provided inaccurate information about their weight and height, which affects the accuracy of the BMI. In this respect, objective measures in future studies is recommended. While adolescents who were physically active or passive or passerby on UGSs were recruited and interviewed, the selection could have biased the sample. Therefore, it might represent a biased sample. Lastly, even though the regression models were significant, the low R squared value suggests that there are other factors that influence adolescents’ GE, general health, and BMI. Hence, the regression models do not completely predict adolescent’ GE, general health, and BMI. Before any strong conclusion can be made, further studies are needed.

## 5. Conclusions

Urbanization has been reducing outdoor play and recreational areas for adolescents, while low and/or declining levels of PA and the increasing rate of adiposity have been reported in studies. As studies show, the condition of the living environment significantly affects adolescents’ PA behavior and GE [27]. Therefore, keeping and managing green infrastructure in cities is not only important for environment but it is also important for human health. Green infrastructure has the potential to reduce health risks in terms of mental and physical health and well-being caused by climate change [77,78]. In cities, UGSs are important part of the green infrastructure. Hence, it is crucial to identify park features that could encourage or discourage adolescents for engaging in GE. Furthermore, it is up to policy makers, urban planners, and landscape architects create UGSs that address adolescents’ needs and encourage them to engage in GE. The findings of this study reveal that distance to UGSs, many trees, lawns, flowerbeds, open areas, exercise trails, play and outdoor fitness equipment, and soccer fields and basketball courts are significantly associated with adolescents’ GE, general health, and BMI. The findings of this paper could be used for policy makers and landscape architects to promote science-based decision making and priority setting when creating, designing, or renewing UGSs. The findings also indicate that adolescents of different gender vary widely in the use of characteristics of UGSs. Therefore, gender differences need to be investigated in future studies. It is also recommended that age differences should also be considered when investigating the use of type of activity settings in UGSs.

## Figures and Tables

**Table 1:** The status of adolescents’ green exercise (GE) and screen time (*n* = 384).

Variables		Overall	Boys (55.2%)	Girls (44.8%)
Frequency of GE	Seldom or never:	15.4%	17.0%	13.4%
	1–2 times a month:	31.5%	32.2%	33.1%
	Weekly:	20.8%	20.3%	21.5%
	Several times per week:	22.7%	24.0%	20.9%
	Daily:	9.6%	8.5%	11.0%
Duration of GE	Less than 15 min:	15.6%	15.6%	15.7%
	15–30 min:	24.0%	23.6%	24.4%
	30 min–1 h:	31.3%	26.9%	36.6%
	1–2 h:	21.1%	22.6%	19.2%
	More than 2 h:	8.1%	11.3%	4.1%
Screen Time	Less than 30 min:	14.3%	12.7%	16.3%
	30 min–1 h:	17.4%	12.7%	23.3%
	1–2 h:	21.4%	20.3%	22.7%
	2–3 h:	20.8%	20.3%	21.5%
	3–4 h:	12.0%	15.6%	7.6%
	More than 4 h:	14.1%	18.4%	8.7%

**Table 2:** Associations between characteristics of urban green spaces (UGSs) and adolescents’ frequency and duration of GE.

Variables	Frequency of GE		Duration of GE	
	*b*	SE	*b*	SE
Gender (boys)	−0.054	0.131	0.216 *	**0.126**
Age	0.101	0.057	0.056	0.055
Education	−0.582 **	**0.209**	−0.112	0.201
Income	0.038	0.044	0.117 **	**0.043**
Body mass index (BMI)	−0.018	0.021	−0.038 *	**0.020**
Screen time	−0.028	0.040	−0.012	0.039
UGSs distance	−0.117 **	**0.037**	−0.004	0.035
Many trees	0.975 *	**0.532**	0.339	0.512
Lawns	1.096 *	**0.582**	−0.335	0.560
Lights	−1.013	0.678	−0.233	0.653
Exercise trails	0.191	0.155	0.097	0.149
Play equipment	1.609 *	**0.926**	0.550	0.891
Soccer field and basketball court	0.293 *	**0.157**	0.183	0.151
Varied plants and animal life	−0.024	0.105	0.017	0.101
Open areas	−0.529	0.620	0.201	0.596
Flowerbeds	−0.083	0.328	0.097	0.316
Outdoor fitness equipment	−0.045	0.149	0.135	0.143
Many benches/seating	0.194	0.275	−0.002	0.264
Picnic areas	0.085	0.209	−0.137	0.201
R^2^	0.108 **		0.063 *	

Note: * *p* ≤ 0.05, ** *p* ≤ 0.01. *b*: Unstandardized coefficients, SE = standard error. Bold indicates the relationship is significant. Girls are the reference group.

**Table 3:** Associations between characteristics of UGSs and adolescents’ self-reported general health and BMI.

Variables	General Health		BMI	
	*b*	SE	*b*	SE
Gender (boys)	0.162	0.111	1.022 ***	**0.318**
Age	−0.073	0.049	0.168	0.140
Education	0.447 *	**0.178**	0.940 *	**0.513**
Income	0.025	0.038	0.017	0.110
Screen time	−0.037	0.034	−0.050	0.098
Park distance	−0.015	0.032	0.112	0.091
Frequency of GE	−0.053	0.049	−0.010	0.141
Duration of GE	0.161 **	**0.051**	−0.238	0.146
Many trees	−0.689	0.452	−2.208 *	**1.300**
Lawns	0.824 *	**0.495**	−2.102 **	**1.425**
Lights	1.075	0.575	2.981	1.655
Exercise trails	0.319 *	**0.132**	−0.569 **	**0.379**
Play equipment	1.324	0.786	−3.870	2.262
Soccer field and basketball court	−0.047	0.134	−0.171	0.386
Varied plants and animal life	0.008	0.089	0.342	0.255
Open areas	0.333	0.521	−4.099	1.501
Flowerbeds	0.088	0.276	2.138	0.795
Outdoor fitness equipment	−0.026	0.125	−1.021 **	**0.361**
Many benches/seating	−0.145	0.231	1.744 **	**0.666**
Picnic areas	−0.153	0.175	1.599 **	0.504
R^2^	0.077 **		0.118 ***	

Note: * *p* ≤ 0.05, ** *p* ≤ 0.01, *** *p* ≤ 0.001. *b*: Unstandardized coefficients, SE = standard error. Bold indicates the relationship is significant. Girls are the reference group.

**Table 4:** Associations between characteristics of UGSs and adolescents’ GE, stratified by gender.

Variables	Boys				Girls			
	Frequency of GE		Duration of GE		Frequency of GE		Duration of GE	
	*b*	SE	*b*	SE	*b*	SE	*b*	SE
Age	0.132 *	**0.075**	0.080	0.076	0.055	0.092	0.013	0.083
Education	−0.544 *	**0.285**	−0.250	0.287	−0.633 **	**0.321**	0.067	0.289
Income	0.025	0.055	0.111 **	**0.055**	0.034	0.084	0.124 *	**0.076**
BMI	−0.043 *	**0.032**	−0.041	0.033	−0.002	0.029	−0.033	0.026
Screen time	−0.090 *	**0.053**	−0.029	0.053	0.053	0.065	0.013	0.059
Park distance	−0.099	0.051	0.025	0.051	−0.159 ***	**0.055**	−0.060	0.050
Many trees	0.766	0.690	0.420	0.695	1.413	0.868	0.304	0.782
Lawns	−0.987	0.769	−0.450	0.775	−1.356	0.932	−0.308	0.839
Lights	−1.052	0.901	−0.466	0.909	−1.061	1.078	−0.091	0.971
Exercise trails	0.250	0.213	0.209	0.215	0.130	0.237	−0.008	0.213
Play equipment	1.622	1.223	0.968	1.233	1.760	1.480	0.172	1.332
Soccer fields and basketball courts	0.150	0.206	0.160	0.208	0.516 **	**0.253**	0.212	0.228
Varied plants and animal life	−0.111	0.136	−0.069	0.137	0.116	0.170	0.124	0.153
Open areas	−0.574	0.805	−0.003	0.812	−0.588	1.010	0.455	0.909
Flowerbeds	−0.150	0.428	−0.082	0.431	−0.043	0.533	0.353	0.480
Outdoor fitness equipment	−0.068	0.199	0.107	0.201	−0.041	0.235	0.184	0.211
Many benches/seating	0.282	0.362	0.204	0.365	0.128	0.440	−0.286	0.396
Picnic areas	0.120	0.274	−0.067	0.276	0.089	0.337	−0.223	0.304
R^2^	0.082		0.079		0.103 *		0.055	

Note: * *p* ≤ 0.05, ** *p* ≤ 0.01, *** *p* ≤ 0.001. *b*: Unstandardized coefficients, SE = standard error. Bold indicates the relationship is significant.

**Table 5:** Associations between characteristics of UGSs and adolescents’ self-reported general health and BMI, stratified by gender.

Variables	Boys				Girls			
	General Health		BMI		General Health		BMI	
	*b*	SE	*b*	SE	*b*	SE	*b*	SE
Age	−0.121 *	**0.066**	214	0.165	−0.008	0.077	0.172	0.248
Education	0.516 **	**0.249**	0.860	0.624	0.295	0.270	1.111	0.874
Income	0.020	0.048	−0.165	0.120	0.040	0.070	0.317	0.227
Screen time	−0.036	0.046	−0.202 *	**0.115**	−0.026	0.054	0.174	0.176
Park distance	0.017	0.045	−0.021	0.113	−0.053	0.047	0.226	0.152
Frequency of GE	−0.053	0.072	−0.143	0.181	−0.063	0.069	0.078	0.225
Duration of GE	0.173 **	**0.071**	−0.115	0.179	0.132 *	**0.077**	−0.323	0.249
Many trees	−0.530	0.601	−0.094	1.510	−0.945	0.715	−3.671 **	**1.319**
Lawns	0.560	0.671	−0.050	1.684	1.229	0.768	−3.661 **	**1.490**
Lights	0.620	0.786	0.440	1.974	1.738 *	**0.883**	4.140	2.864
Exercise trails	−0.229	0.186	−0.084	0.466	0.453 **	**0.195**	−1.316 **	**0.631**
Play equipment	−0.777	1.067	−0.238	2.680	2.149 **	**1.214**	−3.697 **	**0.936**
Soccer field and basketball court	−0.076	0.179	0.080	0.451	−0.040	0.212	−0.840	0.689
Varied plants and animal life	−0.119	0.118	0.150	0.297	0.189	0.141	0.680	0.458
Open areas	−0.256	0.700	1.179	10.758	1.130	0.817	−2.529 ***	**2.648**
Flowerbeds	−0.220	0.371	0.858	0.932	0.514	0.433	−1.139 ***	**1.404**
Outdoor fitness equipment	−0.178	0.173	0.317	0.435	0.165	0.190	−0.987 ***	**0.616**
Many benches/seating	0.106	0.315	−0.513	0.790	−0.496	0.357	−3.529	1.156
Picnic areas	0.055	0.238	−0.541	0.598	−0.420	0.271	−3.111	0.879
R^2^	0.082		0.100 *		0.096 *		0.138 **	

Note: * *p* ≤ 0.05, ** *p* ≤ 0.01, *** *p* ≤ 0.001. *b*: Unstandardized coefficients, SE = standard error. Bold indicates the relationship is significant.

## References

[1] Bakanlığı S. (2014). Türkiye Beslenme ve Sağlık Araştırması 2010: Beslenme Durumu ve Alışkanlıklarının Değerlendirilmesi Sonuç Raporu (Turkey Nutrition and Health Survey 2010: Status and Assessment of Nutritional Habits Final Report).

[2] Şık B. (2017). Çocukluk Çağı Obezitesi Raporu.

[3] Lee I.-M., Shiroma E.J., Lobelo F., Puska P., Blair S.N., Katzmarzyk P.T., Lancet Physical Activity Series Working Group (2012). Effect of physical inactivity on major non-communicable diseases worldwide: An analysis of burden of disease and life expectancy. Lancet.

[4] Guthold R., A Stevens G., Riley L.M., Bull F.C. (2018). Worldwide trends in insufficient physical activity from 2001 to 2016: A pooled analysis of 358 population-based surveys with 1·9 million participants. Lancet Glob. Health.

[5] Hallal P.C., Victora C.G., Azevedo M.R., Wells J.C.K. (2006). Adolescent physical activity and health: A systematic review. Sports Med..

[6] Reilly J.J., Kelly J. (2011). Long-term impact of overweight and obesity in childhood and adolescence on morbidity and premature mortality in adulthood: Systematic review. Int. J. Obes..

[7] Warburton D.E., Nicol C.W., Bredin S.S. (2006). Health benefits of physical activity: The evidence. Can. Med Assoc. J..

[8] WHO (2010). Global Recommendations on Physical Activity for Health.

[9] Gunter K.B., Almstedt H.C., Janz K.F. (2012). Physical activity in childhood may be the key to optimizing lifespan skeletal health. Exerc. Sport Sci. Rev..

[10] Janssen I., LeBlanc A.G. (2010). Review Systematic review of the health benefits of physical activity and fitness in school-aged children and youth. Int. J. Behav. Nutr. Phys. Act..

[11] The Ministry of Health (2014). Physical Activity Guidelines for Turkey.

[12] Riethmuller A.M., Jones R.A., Okely A.D. (2009). Efficacy of Interventions to Improve Motor Development in Young Children: A Systematic Review. Pediatrics.

[13] Fisher A., Boyle J.M., Paton J.Y., Tomporowski P., Watson C., McColl J.H., Reilly J.J. (2011). Effects of a physical education intervention on cognitive function in young children: Randomized controlled pilot study. Pediatrics.

[14] Sibley B.A., Etnier J.L. (2003). The Relationship between Physical Activity and Cognition in Children: A Meta-Analysis. Pediatr. Exerc. Sci..

[15] Kellert S.R. (2005). Nature and childhood development. Building for Life: Designing and Understanding the Human-Nature Connection.

[16] Philbrook L.E., El-Sheikh M. (2016). Associations between neighborhood context, physical activity, and sleep in adolescents. Sleep Health.

[17] Dencker M., Thorsson O., Karlsson M., Lindén C., Eiberg S., Wollmer P., Andersen L. (2006). Daily physical activity related to body fat in children aged 8-11 years. J. Pediatr..

[18] Hills A., Andersen L.B., Byrne N.M. (2011). Physical activity and obesity in children. Br. J. Sports Med..

[19] Dadvand P., Villanueva C.M., Font-Ribera L., Martinez D., Basagaña X., Belmonte J., Vrijheid M., Grazuleviciene R., Kogevinas M., Nieuwenhuijsen M.J. (2014). Risks and Benefits of Green Spaces for Children: A Cross-Sectional Study of Associations with Sedentary Behavior, Obesity, Asthma, and Allergy. Environ. Health Perspect..

[20] Motl R.W., Birnbaum A.S., Kubik M.Y., Dishman R.K. (2004). Naturally occurring changes in physical activity are inversely related to depressive symptoms during early adolescence. Psychosom. Med..

[21] Strong W.B., Malina R.M., Blimkie C.J., Daniels S.R., Dishman R.K., Gutin B., Hergenroeder A.C., Must A., Nixon P.A., Pivarnik J.M. (2005). Evidence Based Physical Activity for School-age Youth. J. Pediatr..

[22] Brown H., Pearson N., Braithwaite R., Brown W., Biddle S. (2012). Physical activity interventions and depression in children and adolescents: A systematic review and meta-analysis. J. Sci. Med. Sport.

[23] Rasberry C.N., Lee S.M., Robin L., Laris B., Russell L.A., Coyle K.K., Nihiser A.J. (2011). The association between school-based physical activity, including physical education, and academic performance: A systematic review of the literature. Prev. Med..

[24] Ekeland E., Heian F., Hagen K.B., Abbott J.M., Nordheim L. (2004). Exercise to improve self-esteem in children and young people. Cochrane Database Syst. Rev..

[25] Kirk D. (2005). Physical education, youth sport and lifelong participation: the importance of early learning experiences. Eur. Phys. Educ. Rev..

[26] Jose K.A., Blizzard L., Dwyer T., McKercher C., Venn A.J. (2011). Childhood and adolescent predictors of leisure time physical activity during the transition from adolescence to adulthood: A population based cohort study. Int. J. Behav. Nutr. Phys. Act..

[27] Sallis J.F., Cervero R.B., Ascher W., Henderson K.A., Kraft M.K., Kerr J. (2006). An ecological approach to creating active living communities. Annu. Rev. Public Health.

[28] Sallis J.F., Owen N., Fisher E.B. (2008). Ecological models of health behavior. Health Behavior and Health Education: Theory, Research, and Practice.

[29] Magalhães A.P., Pina M.F., Ramos E.D. (2017). The Role of Urban Environment, Social and Health Determinants in the Tracking of Leisure-Time Physical Activity throughout Adolescence. J. Adolesc. Health.

[30] Gardsjord H.S., Tveit M.S., Hordh H. (2014). Promoting Youth’s Physical Activity through Park Design: Linking Theory and Practice in a Public Health Perspective. Landsc. Res..

[31] Akpinar A. (2017). Urban green spaces for children: A cross-sectional study of associations with distance, physical activity, screen time, general health, and overweight. Urban For. Urban Green..

[32] Zhang R., Wulff H., Duan Y., Wagner P. (2018). Associations between the physical environment and park-based physical activity: A systematic review. J. Sport Health Sci..

[33] Bloemsma L.D., Gehring U., Klompmaker J.O., Hoek G., Janssen N.A., Smit H.A., Vonk J.M., Brunekreef B., Lebret E., Wijga A.H. (2018). Green Space Visits among Adolescents: Frequency and Predictors in the PIAMA Birth Cohort Study. Environ. Health Perspect..

[34] An R., Shen J., Yang Q., Yang Y. (2019). Impact of built environment on physical activity and obesity among children and adolescents in China: A narrative systematic review. J. Sport Health Sci..

[35] Epstein L.H., Raja S., Gold S.S., Paluch R.A., Pak Y., Roemmich J.N. (2006). Reducing sedentary behavior: The relationship between park area and the physical activity of youth. Psychol. Sci..

[36] McCurdy L.E., Winterbottom K.E., Mehta S.S., Roberts J.R. (2010). Using nature and outdoor activity to improve children’s health. Curr. Probl. Pediatr. Adolesc. Health Care.

[37] Chawla L. (2015). Benefits of Nature Contact for Children. J. Plan. Lit..

[38] Tillmann S., Tobin D., Avison W., Gilliland J. (2018). Mental health benefits of interactions with nature in children and teenagers: A systematic review. J. Epidemiol. Community Health.

[39] Barton J., Pretty J. (2010). What is the Best Dose of Nature and Green Exercise for Improving Mental Health? A Multi-Study Analysis. Environ. Sci. Technol..

[40] Thompson C.J., Boddy K., Stein K., Whear R., Barton J., Depledge M.H. (2011). Does participating in physical activity in outdoor natural environments have a greater effect on physical and mental wellbeing than physical activity indoors? A systematic review. Environ. Sci. Technol..

[41] Gladwell V.F., Brown D.K., Wood C., Sandercock G.R., Barton J.L. (2013). The great outdoors: How a green exercise environment can benefit all. Extrem. Physiol. Med..

[42] Duncan M.J., Clarke N.D., Birch S.L., Tallis J., Hankey J., Bryant E., Eyre E.L.J. (2014). The Effect of Green Exercise on Blood Pressure, Heart Rate and Mood State in Primary School Children. Int. J. Environ. Res. Public Health.

[43] McCormick R. (2017). Does Access to Green Space Impact the Mental Well-being of Children: A Systematic Review. J. Pediatr. Nurs..

[44] Tesler R., Plaut P., Endvelt R. (2018). The Effects of an Urban Forest Health Intervention Program on Physical Activity, Substance Abuse, Psychosomatic Symptoms, and Life Satisfaction among Adolescents. Int. J. Environ. Res. Public Health.

[45] Cohen D.A., Ashwood J.S., Scott M.M., Overton A., Evenson K.R., Staten L.K., Porter D., McKenzie T.L., Catellier D., Ashwood S. (2006). Public parks and physical activity among adolescent girls. Pediatrics.

[46] Wolch J., Jerrett M., Reynolds K., McConnell R., Chang R., Brady K., Gilliland F., Su J.G., Berhane K. (2011). Childhood obesity and proximity to urban parks and recreational resources: A longitudinal cohort study. Health Place.

[47] Wang J.-J., Wang M., Lau P.W., Ainsworth B.E., He G., Gao Y. (2018). Physical activity as a mediator of the associations between perceived environments and body mass index in Chinese adolescents. Health Place.

[48] Van Hecke L., Deforche B., Van Dyck D., De Bourdeaudhuij I., Veitch J., Van Cauwenberg J. (2016). Social and Physical Environmental Factors Influencing Adolescents’ Physical Activity in Urban Public Open Spaces: A Qualitative Study Using Walk-Along Interviews. PLoS ONE.

[49] Edwards N., Hooper P., Knuiman M., Foster S., Giles-Corti B. (2015). Associations between park features and adolescent park use for physical activity. Int. J. Behav. Nutr. Phys. Act..

[50] Dunton G.F., Almanza E., Jerrett M., Wolch J., Pentz M.A. (2014). Neighborhood park use by children: Use of accelerometry and global positioning systems. Am. J. Prev. Med..

[51] Lloyd K., Burden J., Kiewa J. (2008). Young girls and urban parks: Planning for transition through adolescence. J. Park Recreat. Adm..

[52] Baran P.K., Smith W.R., Moore R.C., Floyd M.F., Bocarro J.N., Cosco N.G., Danninger T.M. (2014). Park use among youth and adults: Examination of individual, social, and urban form factors. Environ. Behav..

[53] Van Hecke L., Ghekiere A., Van Cauwenberg J., Veitch J., De Bourdeaudhuij I., Van Dyck D., Clarys P., Van De Weghe N., Deforche B. (2018). Park characteristics preferred for adolescent park visitation and physical activity: A choice-based conjoint analysis using manipulated photographs. Landsc. Urban Plan..

[54] Ries A.V., Gittelsohn J., Voorhees C.C., Roche K.M., Clifton K.J., Astone N.M. (2008). The Environment and Urban Adolescents’ Use of Recreational Facilities for Physical Activity: A Qualitative Study. Am. J. Health Promot..

[55] Veitch J., Salmon J., Parker K., Bangay S., Deforche B., Timperio A. (2016). Adolescents’ ratings of features of parks that encourage park visitation and physical activity. Int. J. Behav. Nutr. Phys. Act..

[56] Ruiz-Trasserra A., Pérez A., Continente X., O’Brien K., Bartroli M., Teixidó-Compaño E., Espelt A., Bartroli M. (2017). Patterns of physical activity and associated factors among teenagers from Barcelona (Spain) in 2012. Gac. Sanit..

[57] Kabisch N., Qureshi S., Haase D. (2015). Human–environment interactions in urban green spaces—A systematic review of contemporary issues and prospects for future research. Environ. Impact Assess. Rev..

[58] Besenyi G.M., Kaczynski A.T., Stanis S.A.W., Bergstrom R., Oestman K.B., Colabianchi N. (2016). Gender Differences in the Relationship between Park Proximity and Features and Child and Youth Physical Activity. Child. Youth Environ..

[59] (2013). Aydın Büyükşehir Belediyesi Nazım İmar Planı.

[60] Schipperijn J., Bentsen P., Troelsen J., Toftager M., Stigsdotter U.K. (2013). Associations between physical activity and characteristics of urban green space. Urban For. Urban Green..

[61] Akpinar A., Cankurt M. (2017). How are characteristics of urban green space related to levels of physical activity: Examining the links. Indoor Built Environ..

[62] CDC About Child & Teen BMI. https://www.cdc.gov/healthyweight/assessing/bmi/childrens_bmi/about_childrens_bmi.html.

[63] TUIK (2018). Gelir ve Yaşam Koşulları Araştırması Bölgesel Sonuçları. Türkiye İstatistik Kurumu. http://www.tuik.gov.tr/PreHaberBultenleri.do?id=30756.

[64] Skar M., Wold L.C., Gundersen V., O’Brien L. (2016). Why do children not play in nearby nature? Results from a Norwegian survey. J. Adventure Educ. Outdoor Learn..

[65] Gobster P.H., Westphal L.M. (2004). The human dimensions of urban greenways: Planning for recreation and related experiences. Landsc. Urban Plan..

[66] Hashim N.H.M., Thani S.K.S.O., Jamaludin M.A., Yatim N.M. (2016). A perceptual study on the influence of vegetation design towards women’s safety in public park. Procedia Soc. Behav. Sci..

[67] Granger E., Di Nardo F., Harrison A., Patterson L., Holmes R., Verma A. (2017). A systematic review of the relationship of physical activity and health status in adolescents. Eur. J. Public Health.

[68] Bell J.F., Wilson J.S., Liu G.C. (2008). Neighborhood greenness and 2-year changes in body mass index of children and youth. Am. J. Prev. Med..

[69] Almanza E., Jerrett M., Dunton G., Seto E., Pentz M.A. (2012). A study of community design, greenness, and physical activity in children using satellite, GPS and accelerometer data. Health Place.

[70] Yildiz M., Eldeleklioglu J. (2018). Investigation of the school dropout problem at level of primary and secondary schools in Turkey. Eur. J. Educ. Stud..

[71] YÖK (2019). 2018 YKS: Yükseköğretime Geçişte Il-Bölge Başarıları ve Nüfus Hareketliliği.

[72] Troiano R.P., Berrigan D., Dodd K.W., Mâsse L.C., Tilert T., McDowell M. (2008). Physical Activity in the United States Measured by Accelerometer. Med. Sci. Sports Exerc..

[73] Soga M., Yamanoi T., Tsuchiya K., Koyanagi T.F., Kanai T. (2018). What are the drivers of and barriers to children’s direct experiences of nature?. Landsc. Urban Plan..

[74] Smith A.L., Troped P.J., McDonough M.H., DeFreese J.D. (2015). Youth perceptions of how neighborhood physical environment and peers affect physical activity: A focus group study. Int. J. Behav. Nutr. Phys. Act..

[75] McCormack G.R., Rock M., Toohey A.M., Hignell D. (2010). Characteristics of urban parks associated with park use and physical activity: A review of qualitative research. Health Place.

[76] Veitch J., Salmon J., Ball K. (2007). Children’s Perceptions of the Use of Public Open Spaces for Active Free-play. Child. Geogr..

[77] Coutts C. (2016). Green Infrastructure and Public Health.

[78] Bowen K.J., Lynch Y. (2017). The public health benefits of green infrastructure: the potential of economic framing for enhanced decision-making. Curr. Opin. Environ. Sustain..

